# The effect of hormone therapy on quality of life and breast cancer risk after risk-reducing salpingo-oophorectomy: a systematic review

**DOI:** 10.1186/s12905-017-0370-6

**Published:** 2017-03-21

**Authors:** Tasneem Siyam, Sue Ross, Sandra Campbell, Dean T. Eurich, Nesé Yuksel

**Affiliations:** 1 Faculty of Pharmacy and Pharmaceutical Sciences, University of Alberta, Edmonton, AB T6G 1C9 Canada; 2 Cavarzan Chair in Mature Women’s Health Research, Department of Obstetrics and Gynecology, Rm 5S131 Lois Hole Hospital/Robbins Pavilion Royal Alexandra Hospital, 10240 Kingsway Ave, Edmonton, AB T5H 3V9 Canada; 3 2K4.01 WC Mackenzie Health Science Center, University of Alberta, Edmonton, AB T6G 2R7 Canada; 4 School of Public Health, 2-040 Li Ka Shing HRIF, University of Alberta, Edmonton, AB T6G 2E1 Canada

**Keywords:** Hormone Therapy, BRCA1/2, RRSO, Breast cancer, QOL

## Abstract

**Background:**

It is unclear if the use of hormone therapy (HT) in carriers of BRCA mutations improves the quality of life (QOL) without increasing the risk of breast cancer following a risk-reducing salpingo-oophorectomy (RRSO). Our objective was to assess the effect of HT on QOL and breast cancer risk, after RRSO.

**Methods:**

We searched MEDLINE, EMBASE, CINHAL, and others, from inception to July 22, 2016, to identify relevant studies. Two reviewers independently screened identified records for controlled trials and observational studies that addressed the effect of HT on QOL and breast cancer risk in women with BRCA mutations, post RRSO. Two reviewers independently extracted data on populations, interventions, comparators, outcomes, and methodological quality. Studies addressing the same outcome were synthesized using written evidence summaries or tables.

**Results:**

Of the 1,059 records identified, 13 met our inclusion criteria. All studies were observational. Six studies assessed the effect on QOL. Of these, 3 showed improvement in QOL with HT use. The risk of breast cancer was evaluated in 4 studies. The mean duration of follow-up was 2.6 years (range 0.1-19.1). The risk of breast cancer did not change with HT use in all 4 studies.

**Conclusions:**

Cumulative evidence from our review suggests that short-term HT use following RRSO improves QOL. The effect on breast cancer risk is still unclear. There are too few long-term studies to draw any strong conclusions. The need for future well-designed RCTs for more established evidence is imperative.

**Electronic supplementary material:**

The online version of this article (doi:10.1186/s12905-017-0370-6) contains supplementary material, which is available to authorized users.

## Background

BRCA mutations are associated with an increased risk of breast and ovarian cancer. In women with mutations of BRCA1 genes, the average cumulative risk for breast cancer by age 80 years is 67% and for ovarian cancer 45% [1–4]. In BRCA2 carriers, the average cumulative risks are 66% and 12%, respectively [1–4]. Risk-reducing saplingo-oophorectomy (RRSO) offers reduction in the risk of ovarian cancer of approximately 80%, among BRCA1 and 2 carriers, and of 50% for breast cancer [5]. However, more recent evidence suggests that breast cancer-risk reduction with RRSO may not be significant, particularly for BRCA1 carriers [6, 7]. Since cancer risk estimates for BRCA carriers are age-dependent and tend to be higher in younger age populations, [7] current guidelines recommend RRSO for BRCA carriers before age 40 years or after completion of child-bearing [8–11].

An immediate consequence of RRSO in premenopausal women is surgical menopause. Surgical menopause is associated with symptoms that can significantly affect a woman's quality of life (QOL), including vasomotor and urogenital symptoms, sexual dysfunction, sleep disturbances, and mood changes [12]. Furthermore, these women are at risk of long-term sequelae such as osteoporosis, cardiovascular diseases, and cognitive impairment [13–15]. In women with early menopause, who have no contraindications to hormone therapy (HT), current guidelines recommend the use of HT until the average age of menopause [16–18]. As BRCA mutation carriers would ideally undertake RRSO at an earlier age than women who perform it for other benign reasons or who go through early natural menopause, guidelines specific to BRCA mutation carriers suggest the consideration of short-term HT use due to the unknown nature of long-term safety [10].

The concern in women with BRCA mutations is that HT may further increase breast cancer risk following a RRSO. The Women's Health Initiative (WHI) randomized trials found an increased risk of breast cancer with estrogen plus progestin, although not with estrogen alone [19]. Data from short-term observational studies assessing the risk of breast cancer with HT use after RRSO are inconsistent, and at this time it is unclear if HT increases breast cancer risk following a RRSO [20, 21].

Carriers of BRCA mutations and women at high risk for breast cancer are often challenged by the decision to undertake RRSO due to the health consequences associated with surgical menopause, and the need for HT that may further increase their breast cancer risk. In 2014, Marchetti et al addressed this important topic in a narrative review, but the lack of details of the literature review method lead to concern about the rigor and completeness of the review [22]. Similarly, in early 2016, Birrer et al published a review of evidence about the safety of HT in women with BRCA mutations [23]. Even though they reported in their title and methods that they conducted a systematic review, the study lacked the main elements of a systematic review, such as a comprehensive literature search, an assessment of the methodological quality of studies included, and transparency in reporting the methods and findings [23].

We, therefore, performed a systematic review to assess the effect of HT on QOL and breast cancer risk in women who have BRCA mutations and who also underwent RRSO for breast and ovarian cancer-risk reduction. The effect of HT on other short and long-term outcomes was also evaluated.

## Methods

Our study was designed and conducted in accordance with the guidelines for Meta-Analyses and Systematic Reviews of Observational Studies (​MOOSE) [24].

### Eligibility criteria

Eligible studies included women who had BRCA1/2 mutations or who had a high risk of breast and ovarian cancer (as defined by the original study authors) but had not undergone genetic testing, and who had undergone RRSO for cancer-risk reduction. Studies comparing the effect of HT (with no restriction on type, dose, regimen, or route of administration) to placebo, non-exposed group or baseline, qualified for inclusion. All controlled trials and observational studies (including prospective and retrospective cohort studies, case-control studies, and cross-sectional studies) were included. Review papers were screened for cited articles. Exclusion criteria included qualitative studies, hypothetical decision analysis, editorials and studies that did not assess the effect of HT on outcomes of interest. Studies that included women with a personal history of breast cancer were not explicitly excluded.

### Outcome measures

Primary outcomes were QOL (general and menopause-specific) and breast cancer risk. Secondary outcomes included: vasomotor symptoms, vulvovaginal atrophy (VVA), sexual function, mood, sleep disturbance, bone loss, cardiovascular disease, stroke, venous thromboembolism, and mortality.

### Data sources and search strategy

A systematic literature search was conducted by a librarian (SC) to identify all relevant published and unpublished studies. Searches using both controlled vocabulary and natural language were performed in databases including MEDLINE (1946 to March 7, 2016), EMBASE (1974 to March 7, 2016), and CINHAL (inception to March 7, 2016) (Additional file 1). Natural language search terms were derived from three main concepts: 1) RRSO, 2) BRCA mutations or high risk of breast and ovarian cancer, and 3) HT. Grey literature searches were conducted in SCOPUS, Web of Science, Google Scholar, Proquest, Dissertations and Theses and clinical trials registries, from inception to July 22, 2016 (Additional file 2). Other searches included hand searches of the reference list of review papers; and citation search of studies included in the systematic review. To increase the sensitivity of our search no language or date restrictions of publications were applied.

### Study selection

Two-step screening for eligibility was performed independently by 2 reviewers (TS and NY), with disagreements resolved by consensus. First, titles and abstracts were screened to select articles eligible for further review. Second, full-text of relevant articles was reviewed for eligibility. Reviewer agreement for confirmation of eligibility was 100%.

### Data extraction and quality assessment

Data extraction was completed independently by two reviewers (TS, AB), and discrepancies resolved by a third reviewer (NY). Data elements extracted included: manuscript characteristics; study design and settings; population characteristics; interventions; comparators; outcomes; and adjustments for potential confounders. The risk of bias assessment was conducted independently by two reviewers (TS, NY) and discrepancies resolved by consensus. The quality of studies was evaluated using the Jadad scale for RCTs, [25] and relevant versions of the Newcastle-Ottawa scale (NOS) for observational studies [26]. Cut off scores of ≥ 4 for Jadad scale and ≥7 for NOS were used to distinguish study quality [27]. Quality assessment scores were used to inform sensitivity analyses to evaluate its effect on pooled measure(s) of effect. Corresponding authors were contacted when data on outcomes were not available.

### Data synthesis

Outcome data were synthesized by tabulating together all studies reported on specific outcomes. For each study, the outcomes reported were grouped by HT users versus non-users, with mean differences or measures of association as relevant. Descriptive analysis was used for each outcome.

When sufficient homogeneity was demonstrated, outcome data were pooled quantitatively via a meta-analysis (as only two or three papers could be pooled for each outcome variable the details of the meta-analysis can be found in Additional file 3).

## Results

**Fig. 1:**
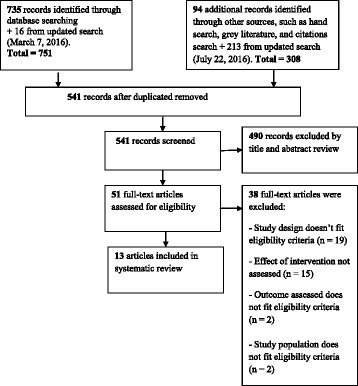
Flow chart for study identification and selection

Our search identified 1,059 records of which 51 full-text articles were retrieved and assessed for eligibility, and 13 were included (Fig. 1.) The most common reasons for exclusion are listed in Fig. 1. ​Additional file 4 lists all 51 studies reviewed for eligibility and the reason for exclusion whenever this may apply.

### Study characteristics

Table 1 lists the main characteristics of the studies and their methodological quality. No RCTs were identified. The mean age of women across studies was 49.87 years (range 33-83), however, 6 studies did not report the participants’ age [21, 28–32]. The mean age at RRSO surgery was 45.54 years (range 24-80). Studies included both BRCA1 and 2 carriers, except for 2, which included only BRCA1 carriers [21, 31]. In addition to BRCA mutation carriers, 6 studies included women who had high risk of breast and ovarian cancer with no confirmed genetic diagnosis [29, 32–36]. Other variables, such as time since RRSO, body mass index (BMI), smoking status, history of breast cancer and hysterectomy were reported in some but not all studies. No studies included women with personal history of breast cancer. Intervention characteristics are listed in Table 2.

**Table 1:** Study characteristics

First author, year of publication	Study design	Sample size	Sample size for RRSO	Age at time of study^a^, mean (range/SD)^b^	BRCA status (%)	Menopause status at time of RRSO (%)	Age at RRSO surgery, mean(range/SD)^b^	Comparator	Quality rating
Challberg, 2011 [33]	Cross-sectional survey design	212	212	50(36-77)	BRCA1 & BRCA2 (58%)	Premenopausal (100%)	41.20 (24-48)	Non-exposed and previous users	Low
Chapman, 2011 [37]	Cross-sectional survey design	51	51	49 (36-54)	BRCA1 (63%) & BRCA2 (37%)	Premenopausal (47%) & Postmenopausal (53%)	46 (31–68)	Non-exposed	Low
Eisen, 2008 [21]	Matched case control study	472	136	NS	BRCA1 (100%)	Premenopausal (100%)	42.45 (28 – 52)^c^	Control (no outcome)	High
Finch, 2011 [38]	Prospective cohort study	114	114	53(42-74)	BRCA1 (51%) & BRCA2 (49%)	Premenopausal (66%) & Postmenopausal (34%)	47.50 (35-69)	Non-exposed, baseline	Low
Gabriel, 2008 [28]	Retrospective cohort study	73	73	NS	BRCA1 (64%) & BRCA2 (38%)	Premenopausal (NS) & Postmenopausal (NS)	42 (29.5-59.2)	Non-exposed	Low
Garcia, 2015 [30]	Retrospective chart review	225	225	NS	BRCA1 & 2 (100%)	Premenopausal (NS) & Postmenopausal (NS)	50 (31-80)	Non-exposed	Low
Heiniger, 2014 [29]	Matched prospective cohort design	233	38	NS	BRCA1 & 2 (16.7%)	Premenopausal (NS) & Postmenopausal (NS)	NS	Non-exposed and previous users	Low
Johansen, 2016 [36]	Retrospective cohort study	1522	294	54(33-83)	NS^d^	Premenopausal (NS) & Postmenopausal (NS)	48(31-76)	Non-exposed	Low
Kotsopoulos, 2016 [31]	Matched case control study	864	210	NS	BRCA 1 (100%)	Premenopausal (NS) & Postmenopausal (NS)	42.75(28-53)^c^	Control (no outcome)	Low
Madalinska, 2006 [34]	Cross-sectional survey design	450	450	46 ± 6 (range34-59)	BRCA1 & 2 (48%)	Premenopausal (100%)	43 ± 6	Non-exposed	High
Michelsen, 2009 [35]	Cross-sectional survey design	1956	326	54.4(8.9)	BRCA1 & 2 (20%)	Premenopausal (NS) & Postmenopausal (NS)	48 ± 7.8	Non-exposed	High
Rebbeck, 2005 [20]	Prospective cohort study	462	155	42.7(37-78)	BRCA1 (70%) & BRCA2 (30%)	Premenopausal (NS) & Postmenopausal (NS)	NS	Non-exposed	High
Tucker, 2016 [32]	Cross-sectional survey design	119	119	NS	BRCA1 (8.40%) & BRCA2 (11.70%)	Premenopausal (43%) & Postmenopausal (57%)	50(33-69)	Non-exposed	Low

*NS* Not specified, *RRSO* Risk-reducing salpingo-oophorectomy

^a^for RRSO cohort only; ^b^based on the measure of variance reported in the primary study; ^c^average age at surgery among cases and controls; ^d^BRCA status is captured in the questionnaire but not reported in paper

**Table 2:** Intervention characteristics

First author	Type of HT	Dose of HT	Route of HT	Duration of HT^a^-mean (range/SD)^b^
Challberg [33]	ET, EPT and tibolone	NS	NS	3.4 (0.1-19)
Chapman [37]	NS	NS	NS	6 (0.75-9)
Eisen [21]	ET and EPT	NS	NS	3.85^c^ (NS)
Finch [38]	ET and EPT	NS	NS	NS
Gabriel [28]	ET and EPT	NS	NS	2.79 ± 3.22
Garcia [30]	NS	NS	Systemic HT (60%)	NS
Heiniger [29]	NS	NS	NS	NS
Johansen [36]	ET, EPT and tibolone	NS	Systemic HT (39.28%) & local/vaginal HT (6.54%)	NS
Kotsopoulos [31]	ET and EPT	NS	NS	4.35(0.05-25)^c^
Madalinska [34]	EPT and tibolone	NS (standard)	Systemic HT (Oral/transdermal)	3 ± 2.3
Michelsen [35]	NS	NS	Systemic (Oral/transdermal)	NS
Rebbeck [20]	ET and EPT	NS	NS	NS
Tucker [32]	ET	NS	Systemic HT (20% - oral and transdermal) & local/vaginal HT (8%)	NS

*HT* hormone therapy, *ET* estrogen therapy, *EPT* estrogen-progestogen therapy, *NS* not specified

^a^in years; ^b^based on the measure of variance reported in the primary study; ^c^average duration of use among cases and controls

### Synthesis of results

The outcomes reported for individual studies are shown in Table 3.

**Table 3:** Outcome data for individual studies: HT users versus non-users

Outcome	First author	Tool of assessment	N of analysis	Mean difference^ab^	Measure of association (95% CI)^c^	*P* value	Duration of follow-up
General QOL	Tucker [32]	SF-36 ​–​ total	108	Systemic HT = 1.76	-	0.57	NA
			93	Local HT = 3.3	-	0.86	
		SF-36 – pain	108	Systemic HT = 14.64	-	**<0.01**	
			93	Local HT = 4.85	-	0.75	
		SF-36 – physical	108	Systemic HT = 7.15	-	0.38	
			93	Local HT = 5.34	-	0.52	
		SF-36 – emotional	108	Systemic HT = -0.50	-	0.50	
			93	Local HT = -5.5	-	0.27	
		SF-36 – social	108	Systemic HT = -3.67	-	0.82	
			93	Local HT = 3.66	-	0.92	
		SF-36 – energy	108	Systemic HT = 0.6	-	0.42	
			93	Local HT = 3.66	-	0.87	
		SF-36 – general health	108	Systemic HT = 4.55	-	0.55	
			93	Local HT = 3.37	-	0.96	
Menopause specific QOL	Challberg [33]	FACT-ES^d^ – total	141	3.1	-	0.09	NA
	Chapman [37]	MSL^e^ – total	51	-1.1	-	0.06	NA
	Finch [38]	MENQOL Intervention^e^ – total	73	-3.37^f^	-	**<0.01**	13.6 months (10.8–21.8)
		MENQOL – vasomotor	73	-3.4	-	**<0.01**	
		MENQOL – physical	73	-0.38	-	0.28	
		MENQOL – psychosocial	73	-0.07	-	0.89	
		MENQOL – sexual	73	-1.22	-	**0.02**	
	Heiniger [29]	MRS^e^	38	NS	-	>0.05	3 years^g^
	Madalinska [34]	FACT-ES^d^ – total	164	3.4	-	**0.03**	NA
	Tucker [32]	MENQOL^e^ – total	108	Systemic HT = -2.76^f^	-	**<0.01**	
			93	Local HT = -2.23^f^	-	**<0.01**	
		MENQOL – vasomotor	108	Systemic HT = -1.08	-	**0.02**	
			93	Local HT = -1.04	-	0.22	
		MENQOL – physical	108	Systemic HT = -0.74	-	**0.03**	
			93	Local HT = -0.54	-	0.38	
		MENQOL – psychosocial	108	Systemic HT = -0.1	-	0.36	
			93	Local HT = -0.1	-	0.91	
		MENQOL – sexual	108	Systemic HT = -0.84	-	**0.03**	
			93	Local HT = -0.55	-	0.74	
Breast cancer	Eisen [21]	Self-reported^h^	124	-	OR = 0.48(0.19-1.21)	0.12	NA
	Kotsopoulos [31] *Same study as Eisen but an updated analysis*	Self-reported	210	-	OR = 1.06(0.58-1.96)	0.85	NA
					OR = 1.06(0.52-2.18) - *Breast cancer risk with HT use of* ≤*3 years* vs. *never use*	0.87	
					OR = 1.06 (0.41-2.71) - *Breast cancer risk with HT use of >3 years* vs. *never use*	0.91	
	Gabriel [28]	Self-reported^h^	60	-	OR = 0.31(0.09-1.04)^f^	>0.05	NS
					OR = 0.48(0.1-2.1) - *Breast cancer risk with ET only (no cases with EPT)*	>0.05	
	Rebbeck [20]	Medical records, operative notes, and pathology reports	155	-	HR = 3.93(0.51-30.50)^i^	>0.05	2.6 years (0.1-19.1)
					HR = 2.56(0.08-78.13) *Breast cancer risk with EPT* vs. *ET*	>0.05	
Vasomotor symptoms	Challberg [33]	FACT-ES^j^	141	-	Hot flashes OR = 0.55(0.23-1.28)^f^	>0.05	NA
					Night sweats OR = 0.28(0.11-0.76)^f^	**<0.05**	
	Finch [38]	Self-reported	73	-	Hot flashes OR = 0.27(0.09-0.80)^f^	**0.03**	13.6 months (10.8–21.8)
	Madalinska [34]	FACT-ES^j^	164	-	Hot flashes OR = 0.34(0.17-0.70)^f^	**<0.01**	NA
					Night sweats OR = 0.51(0.26-1.00)^f^	**0.04**	
Sexual function	Finch^k^ [38]	SAQ^d^	61	Pleasure = 1.22		0.50	13.6 months (10.8–21.8)
				Discomfort = 1.92		**0.03**	
				Habit = 0.19		0.10	
	Heiniger [29]	SAQ^d^	38	NS for all 3 dimensions	-	>0.05	
	Johansen [36]	SAQ^l^	157	Pleasure systemic HT (both ET and EPT) = 0.9	-	>0.05	
			102	Pleasure local HT = -1.5	-	>0.05	
			116	Pleasure systemic ET = 0.8		>0.05	
			111	Pleasure systemic EPT = 0.5		>0.05	
			112	Pleasure systemic tibolone = 1.5		>0.05	
			157	Discomfort systemic HT (both ET and EPT) = -1.2		**<0.01**	
			102	Discomfort local HT = -0.7		0.2	
			116	Discomfort systemic ET = -1.1		**0.04**	
			111	Discomfort systemic EPT = -1.2		**0.02**	
			112	Discomfort systemic tibolone = -1.39		**<0.01**	
	Madalinska [34]	SAQ^d^	164	Pleasure = 0.4		0.70	NA
				Discomfort = 0.4		0.17	
				Habit = 0.1		0.45	
	Tucker [32]	FSFI^dm^ – total	108	Systemic HT 5.36	OR = 0.40(0.12-1.31); *P* = 0.130 *Risk of FSD with systemic HT*	0.14	NA
			93	Local HT 7.61	OR = 0.22(0.05-0.95); *P* = **0.043** *Risk of FSD with local HT*	0.07	
		FSFI – desire^n^	108	Systemic HT 0.09	OR = 0.77(0.23-2.52) *P* = 0.66 *Risk of HSDD with systemic HT*	0.83	
			93	Local HT 0.52	OR = 0.29(0.07-1.28); *P* = 0.10 *Risk of HSDD with local HT*	0.25	
		FSFI – arousal	108	Systemic HT 0.57	-	0.63	
			93	Local HT 1.35	-	0.09	
		FSFI – lubrication^o^	108	Systemic HT 1.39	OR = 0.38(0.12-1.19); *P* = 0.10 *Risk of lubrication difficulty with systemic HT*	**0.04**	
			93	Local HT 1.84	OR = 0.29(0.05-1.53); *P* = 0.14 *Risk of lubrication difficulty with local HT*	**0.03**	
		FSFI – pain^o^	108	Systemic HT 1.97	OR = 0.16(0.03-0.81); *P* = **0.03** *Risk of dyspareunia with systemic HT*	**<0.01**	
			93	Local HT 1.55	OR = 0.99(0.22-4.47); *P* = 0.99 *Risk of dyspareunia with local HT*	0.05	
		FSFI – orgasm^o^	108	Systemic HT 0.71	OR = 0.35(0.10-1.21); *P* = 0.10 *Risk of orgasm difficulty with systemic HT*	0.40	
			93	Local HT 1.47	OR = 0.57(0.10-3.15); *P* = 0.52 *Risk of orgasm difficulty with local HT*	0.13	
		FSFI – satisfaction^o^	108	Systemic HT 0.62	OR = 0.36(0.11-1.14); *P* = 0.08 *Risk of dissatisfaction with sex life with systemic HT*	0.25	
			93	Local HT 0.86	OR = 0.88(0.19-4.06); *P* = 0.87 *Risk of dissatisfaction of sex life with local HT*	0.36	
		FSDS-R^p^	108	Systemic HT -4.07	OR = 0.36(0.16-1.13); *P* = 0.08 *Risk of sexual distress with systemic HT*	0.07	
			93	Local HT -2.34	OR = 1.28(0.30-5.41); *P* = 0.74 *Risk of sexual distress with local HT*	0.94	
Loss of interest in sex	Challberg [33]	FACT-ES^j^	141		OR = 0.68(0.34-1.37)^f^	>0.05	NA
	Madalinska [34]	FACT-ES^j^	164		OR = 0.66(0.30-1.47)^f^	0.35	NA
Vaginal dryness	Challberg [33]	FACT-ES^j^	141		OR = 0.48(0.20-1.16)^f^	>0.05	NA
	Finch [38]	MENQOL Intervention^e^	73	-1.22		**0.02**	13.6 months (10.8–21.8)
	Madalinska [34]	FACT-ES^j^	164	-	OR = 0.47(0.21-1.07)^f^	>0.05	NA
	Tucker^24^	MENQOL – sexual	108	Systemic HT = -0.84	-	**0.03**	
			93	Local HT = -0.55	-	0.74	
		FSFI – lubrication^o^	108	Systemic HT 1.39	OR = 0.38(0.12-1.19); *P* = 0.10 *Risk of lubrication difficulty with systemic HT*	**0.04**	
Bone loss prevention	Challberg[33]		93	Local HT 1.84	OR = 0.29(0.05-1.53); *P* = 0.14 *Risk of lubrication difficulty with local HT*	**0.03**	NA
	Chapman [37]	DXA scan	31	-	OR = 0.41(0.07-2.41)^fi^	>0.05	NA
	Garcia [30]	DXA scan	198		OR = 0.84(0.26-2.74)	>0.05	NA
Cardiovasc-ular disease	Michelsen [35]	Physical measurements, blood samples and self-administered questionnaire	326	-	NS	>0.05	NA

Bold values indicate statistical significance; *CI* confidence interval, *QOL* Quality of life, *SF-36 FACT-ES* 18-item functional assessment of cancer therapy-endocrine score, *NA* Not applicable (due to cross-sectional nature of data), *MSL* menopause symptoms list, *MENQOL* menopause-specific quality of life, *MRS* Menopause rating scale, *SAQ* Sexual activity questionnaire, *FSFI* Female Sexual Function index, *FSD* Female sexual dysfunction, *FSDS-R* Female sexual distress scale- revised, *HSDD* Hypoactive sexual desire disorder

^a^mean score of users minus the mean score of non-users; ^b^continuous outcome; ^c^discrete outcome; ^d^higher score indicates improvement of symptoms; ^e^higher score indicates worsening of symptoms; ^f^measures of effect not reported in primary study but calculated from reported data(unadjusted); ^g^ menopausal symptoms and sexual activity were measured only once in the follow-up interview, no baseline assessment for these variables were performed; ^h^diagnosis confirmed through medical records and pathology reports; ^i^authors contacted for measure of effect and 95% CI as not reported in published paper; ^j^individual symptoms of the FACT-ES scale were dichotomized (symptom present was considered to be a response in either of the two highest categories, “very much” and “quite a bit”); ^k^standard deviation for sexual activity questionnaire domains was not reported in study, values were imputed from Madalinska et al. for meta-analysis[59]; ^l^a higher pleasure score indicates high pleasure and a higher discomfort score indicates higher discomfort; ^m^FSFI- total score is dichotomized to identify risk of FSD with those scoring ≤26.55 considered likely to have FSD; ^n^FSFI-desire sub-score is dichotomized to identify the risk of HSDD with those scoring ≤5 having a high likelihood of HSDD; ^o^dichotomization criterion of these sub-scores was not reported in the primary study; ^p^a cutoff score of ≥11 on the FSDS-R was used to indicate high levels of sexual distress

### Quality of life

Six studies assessed the effect of HT on menopause-specific QOL [29, 32–34, 37, 38]. Tools of QOL assessment varied and included Functional Assessment of Cancer Therapy-Endocrine Score (FACT-ES) [33, 34]; Menopause Symptoms List (MSL) [37], Menopause-Specific Quality of Life-Intervention tool (MENQOL-I), [32, 38] and Menopause Rating Scale (MRS) [29]. Where reported, the mean age of women was 46 years or older in these studies [33, 34, 37, 38]. Studies differed with respect to the menopausal status at the time of RRSO surgery: 4 included pre and postmenopausal women, [29, 32, 37, 38] 2 included only pre-menopausal women [33, 34]. In one study including both pre and postmenopausal women, QOL was analyzed in the pre-menopausal group only [38]. Of the 6 studies evaluating QOL, 3 studies showed improvement in QOL, [32, 34, 38] and 3 showed no change [29, 33, 37]. One study evaluated the effect of HT on general QOL using the Short-form Health Survey (SF-36) [32]. The use of systemic HT improved only the pain domain of the SF-36 survey but none of the other domains.

### Breast cancer

Four studies looked at breast cancer risk with HT use [20, 21, 28, 31]. One study was an update of a previous analysis done by Eisen et al [21, 31]. All 4 studies included women, with confirmed BRCA mutations, of comparable mean age at the time of RRSO surgery and with no personal history of breast cancer. Two studies included BRCA1 and 2 mutations, with BRCA1 carriers, represented ≥60% in both [20, 28]. The remaining 2 studies included only BRCA1 carriers [21, 31]. All studies included ET and EPT users. The mean duration of HT use was 3.83 years (range 0.05-25). The mean duration of follow-up for the only prospective study was 2.6 years (range 0.1-19.1) [20]. Breast cancer risk did not change with HT use in any of the 4 studies.

Only 2 studies reported the effect of HT regimen on breast cancer risk [20, 28]. In Gabriel et al, 3 women on ET developed breast cancer (OR 0.48; 95% CI, 0.1-2.1), with no cases in women on EPT [28]. Rebbeck et al. reported that compared to ET users the risk of breast cancer with EPT was higher but not significant (HR 2.56; 95% CI, 0.08-78.13) [20]. The effect of HT duration of use on breast cancer was reported in one study [31]. Compared to never use, breast cancer risk did not change with greater than 3 years of HT use post RRSO.

### Other outcomes

#### Vasomotor symptoms

Vasomotor symptoms were assessed in 4 studies [32–34, 38]. HT reduced the prevalence and/or severity of hot flashes in all studies.

#### Sexual function

Sexual function was measured in 5 studies as part of the QOL instruments (MENQOL, and FACT-ES), or using the Sexual Activity Questionnaire (SAQ), Female Sexual Function index (FSFI) or Female Sexual Distress Scale – revised (FSDS) [29, 32, 34, 36, 38]. Two studies showed an improvement in sexual function with HT, using the sexual domain of MENQOL (Table 3) [32, 38]. The only aspect of sexual activity that consistently improved with HT use across studies was discomfort/pain [32, 36, 38]. Other aspects of sexual activity, such as pleasure, habit, satisfaction and libido showed no improvement.

#### Vulvovaginal Atrophy (VVA)

Four studies measured the effect on VVA [32–34, 38]. In 2 studies, vaginal dryness was included as a component of sexual function: taking HT improved vaginal dryness and lubrication difficulty with intercourse [32, 38]. Two studies measured the effect of taking HT on VVA, separate from sexual function, and did not find improvement [33, 34].

#### Prevention of bone loss

Three studies evaluated the effect of HT on bone loss [30, 33, 37]. Two studies included the time frame of DXA screening post-RRSO (6.3 years [33] and 1.25 years [30]). HT users had less bone loss compared to non-users in 2 studies [33, 37].

## Discussion

In our rigorously conducted systematic review, women with BRCA mutations who had RRSO had improvements in overall menopause-specific QOL with the use of HT, as well as reduction in vasomotor symptoms and VVA. The association of HT with breast cancer risk is still unclear due to the lack of long-term quality studies.

QOL after RRSO is an important consideration for women who elect to have RRSO. QOL in this population is comparable with the general population, [39, 40] though menopause-specific QOL may be compromised [34, 40–43]. Several studies show that HT improves menopause-specific QOL in symptomatic women who have gone through natural or surgical menopause [44–46]. Our cumulative results are consistent with these findings, as HT improved menopause-specific QOL following RRSO. Although 3 of the studies that assessed QOL showed no change with HT use, findings from these studies were more prone to bias due to confounding than studies that showed improvement in QOL [29, 33, 37].

The risk of breast cancer is the greatest concern women in the general population have when considering HT [47, 48]. This fear stems from the results of the WHI, which showed an increased risk of breast cancer in women on EPT for 5 or more years [19, 49]. These results are often extrapolated to younger surgically menopausal women, even though the WHI participants mean age was 63 years at the time of study recruitment. In contrast, the use of ET alone in the WHI in younger women who have had a hysterectomy showed no increase in breast cancer risk [19, 50, 51]. Unfortunately we were unable to further explore the relative impact of ET versus EPT on breast cancer or other outcomes, as few studies reported the outcomes by specific treatment [20, 21] and not all specified the type of HT [29, 30, 35, 37].

Several recently published narrative reviews evaluated whether HT counteracts the breast cancer risk-reducing effects of RRSO [12, 22, 23, 52]. However, with newer evidence suggesting the lack of breast cancer risk-reducing benefits from RRSO, the clinical inquiry that rather needs to be addressed is whether HT further increases the risk of breast cancer following a RRSO. These review papers concluded that HT seems to be safe in the short-term. We argue that given the scarcity and methodological limitations of the available evidence, no firm conclusions can be drawn, in the short or long term. None of the reviews critically appraised the included studies to assess their risk of bias. In our systematic review, we identified several limitations in the studies assessing breast cancer risk. All studies were affected by recall bias as HT was self-reported. Three of the studies were not designed to capture breast cancer incidence [21, 28, 31]. The only prospective study that captured this outcome had a relatively short follow-up (mean 2.6 years) [20], as well there was selection bias due to lost to follow-up [20]. Furthermore, in this study, breast cancer events within each group were relatively small which may have limited the estimate’s precision and validity. Of note, all studies we identified were in women who had no personal history of breast cancer; we cannot comment on whether the results of our study could be applied to such women.

Among the other outcomes we studied, our systematic review found that HT was associated with a reduction in vasomotor symptoms. The benefits of HT on vasomotor symptoms is already well established [53]. VVA was also shown to improve with systemic HT in the pooled findings from the two relevant studies in our meta-analysis (Additional file 3). This aligns with established evidence in the literature [54]. However, in the individual studies, there was no significant improvement in VVA with HT. Unfortunately information on vaginal estrogen use was not provided in these studies. The one study that reported and evaluated the effect of vaginal estrogen use on vaginal dryness showed a reduction in the severity of the symptom and its risk [32]. Sexual discomfort improved for women taking systemic or local HT, while other sexual dimensions were not found to be significantly different between groups [32, 36, 38]. Sexual function is more complex than hormone levels alone, and other factors such as emotional satisfaction, psychological status, physical health and relationship status also need to be considered [55]. Androgen levels are reduced in surgical menopause [56], and may contribute to low libido [57, 58]. However, in the studies that looked at sexual function in our review, only one study analyzed the effect of androgen levels on sexual desire and arousal and found no association [32]. The effect of testosterone on sexual function was outside the scope of this review.

There are several limitations associated with our study, mainly related to the limitations of the included studies. First, all of the studies included in this review were observational with a small sample size. Evidence from these studies cannot be considered as robust as those from RCTs. Second, very few studies provided sufficient outcome data suitable for meta-analysis limiting the value of these analyses (Additional file 3). Third, several studies in this review did not control for the effect of baseline QOL score and menopause status at the time of RRSO which are considered confounders. The only study that controlled for baseline score showed a significant improvement in QOL with HT [38]. Fourth, we could not assess the effect of HT regimens (ET vs. EPT) on different outcomes as these were poorly reported in most studies. Conclusions from our systematic review may also be affected by publication bias. The preferential publication of studies, with statistically significant treatment effects, may overestimate the effect of HT. Our search strategy aimed to locate both published and unpublished work. We were unable to locate any unpublished efforts.

Despite the limitations, our systematic review possesses several strengths that differentiate it from previous less-structured reviews on this topic [12, 22, 23]. Our review was executed in compliance with MOOSE guidelines (Additional file 5) and based on a pre-specified protocol (PROSPERO registration number: 42014012997). We believe that the rigorous protocol and clear description of our method allow clinicians and RRSO patients to be confident that our findings are as rigorous as they can be based on the relative paucity of good evidence to answer the important questions that RRSO patients are asking.

## Conclusion

Cumulative evidence from our review highlights the benefits of HT in improving QOL and managing common menopausal symptoms induced by RRSO. However, no conclusions can be drawn about the safety of HT, as far as breast cancer risk is concerned. There are too few well-designed long-term studies to draw firm conclusions to guide women and their clinicians in their decision-making about HT. Future well-designed RCTs are needed. In the absence of clear evidence to inform the use of HT post RRSO, clinicians and patients must carefully discuss the potential benefits of HT as well as non-hormonal therapies in improving QOL, in the context of the unknown risk of breast cancer in this population. However, this may not be of concern for women who opt for risk-reducing bilateral mastectomy since the risk of breast cancer in this population is negligible.

## Additional files

Additional file 1: A Search Strategy for Electronic Bibliographic Databases. The document provides a detailed account of the search strategy implemented by an expert searcher (SC) in a variety of electronic databases including MEDLINE, EMBASE, CINAHL, Proquest Dissertations and Theses, SCOPUS, LILACS, PsycINFO, and Cochrane Library. The search was initially performed in February/March 2014 and updated in March 2016. (DOCX 354 kb)
Additional file 2: A Search Strategy for Grey Literature: An Update. The document provides an update of the grey literature search performed in July, 2016, on SCOPUS, Proquest Dissertations and Theses, Clinical trial registries, Web of Science, and Google Scholar. (DOCX 22 kb)
Additional file 3: The Effect of Hormone Therapy on Quality of Life and Breast Cancer Risk After Risk-Reducing Salpingo-oophorectomy: Meta-analyses of Pooled studies. The document provides a written description of the methods and the results of the meta-analyses conducted in our study. (DOCX 39 kb)
Additional file 4: The results of full-text screening of eligible articles and the reason for exclusion whenever this may apply. (DOCX 19 kb)
Additional file 5: A Checklist summarizing compliance with MOOSE guidelines. The attached document is a checklist highlighting the compliance of our systematic review with the quality criteria specified by MOOSE guidelines. (DOC 27 kb)

## Abbreviations

BMI: Body mass index
EPT: Estrogen-progestogen therapy
ET: Estrogen therapy
FACT-ES: Functional Assessment of cancer therapy – endocrine score
FSD: Female sexual dysfunction
FSDS-R: Female sexual distress scale-revised
FSFI: Female sexual function index
HR: Hazard ratio
HSDD: Hypoactive sexual desire disorder
HT: Hormone therapy
MENQOL-I: Menopause-specific quality of life – intervention
MRS: Menopause rating scale
MSL: Menopause symptoms list
OR: Odds ratio
QOL: Quality of life
RCTs: Randomized controlled trials
RRSO: Risk-reducing salpingo-oophorectomy
SAQ: Sexual activity questionnaire
SF-36: Short-form health survey
VVA: Vulvovaginal atrophy
WHI: Women’s health initiative
