# Supplementary material for: The effect of hormone therapy on quality of life and breast cancer risk after risk-reducing salpingo-oophorectomy: a systematic review
Source: BMC Womens Health. 2017 Mar 21;17:22. doi: 10.1186/s12905-017-0370-6 (PMC5359830; doi:10.1186/s12905-017-0370-6)
Supplement: Additional file 1: — A Search Strategy for Electronic Bibliographic Databases. The document provides a detailed account of the search strategy implemented by an expert searcher (SC) in a variety of electronic databases including MEDLINE, EMBASE, CINAHL, Proquest Dissertations and Theses, SCOPUS, LILACS, PsycINFO, and Cochrane Library. The search was initially performed in February/March 2014 and updated in March 2016. (DOCX 354 kb) [file 12905_2017_370_MOESM1_ESM.docx]

**Additional file 1: A Search Strategy for Electronic Bibliographic Databases**

**Methods**

Systematic searches were conducted in a variety of databases including MEDLINE, EMBASE, CINAHL, Proquest Dissertations and Theses, SCOPUS, LILACS, PsycINFO, and Cochrane Library by an expert searcher (SC), between February 25, 2014 and March 21, 2014 and updated in March, 2016. Searches were conducted using controlled vocabularies (eg: MeSH and EMTREE) and key words representing the concepts: (bcra1 or bcra2) and (oopherectomy) and (hormone replacement therap*). No limits were applied. Search strategies are available in Appendix 1.

Grey literature searches were conducted in SCOPUS, Web of Science, Google Scholar, Proquest, Dissertations and Theses and clinical trials registries, between February 25, 2014 and March 21, 2014 and updated in July 2016. Other searches included hand searches of reference list of review papers; and citation search of studies included in the systematic review

**Database: Ovid MEDLINE(R) In-Process & Other Non-Indexed Citations and Ovid MEDLINE(R) <1946 to Present>**
Search Strategy:
--------------------------------------------------------------------------------
1     exp Genes, BRCA2/ or exp Genes, BRCA1/ or [brca1.mp](http://brca1.mp/). (10753)
2     exp BRCA2 Protein/ or [brca2.mp](http://brca2.mp/). (6557)
3     hereditary breast cancer*.mp. (781)
4     (HEREDITARY BREAST and OVARIAN CANCER SYNDROME).mp. [mp=title, abstract, original title, name of substance word, subject heading word, keyword heading word, protocol supplementary concept word, rare disease supplementary concept word, unique identifier] (157)
5     exp BRCA1 Protein/ (3477)
6     "breast cancer 1".mp. (488)
7     "breast cancer 2".mp. (271)
8     genetic [predisposition.mp](http://predisposition.mp/). or exp Genetic Predisposition to Disease/ (93864)
9     exp Breast Neoplasms/ or (ovarian carcinoma* or breast carcinoma* or ovarian cancer* or ovarian neoplasm* or breast cancer* or breast neoplasm*).mp. (314845)
10     8 and 9 (6302)
11     1 or 2 or 3 or 4 or 6 or 7 or 10 (16245)
12     ((family history or familial*) adj1 (ovarian carcinoma* or breast carcinoma* or ovarian cancer* or ovarian
neoplasm* or breast cancer* or breast neoplasm*)).mp. [mp=title, abstract, original title, name of substance word,subject heading word, keyword heading word, protocol supplementary concept word, rare disease supplementary concept word, unique identifier] (1317)
13     11 or 12 (16671)
14     (hormone adj2 therap*).mp. [mp=title, abstract, original title, name of substance word, subject heading word, keyword heading word, protocol supplementary concept word, rare disease supplementary concept word, unique identifier] (26211)
15     exp Hormone Replacement Therapy/ (20628)
16     exp Progesterone Congeners/ or exp Estrogens/ or [hrt.mp](http://hrt.mp/). (180437)
17     exp Progesterone/ or (progesterone adj1 (replacement* or therap*)).mp. (63082)
18     14 or 15 or 16 or 17 (204693)
19     exp Ovariectomy/ (20317)
20     (oophorectom* or ovariectom* or oophorotom* or ovarectom* or ovariotom* or ovarotom* or ovary amputation* or ovary resection* or salpingooophorectom*).mp. [mp=title, abstract, original title, name of substance word, subject heading word, keyword heading word, protocol supplementary concept word, rare disease supplementary concept word, unique identifier] (35698)
21     (female adj castration*).mp. [mp=title, abstract, original title, name of substance word, subject heading word, keyword heading word, protocol supplementary concept word, rare disease supplementary concept word, unique identifier] (27)
22     (BPSO or BOO or BSO or BPO or RRSO).mp. (3518)
23     19 or 20 or 21 or 22 (38969)
24     13 and 18 and 23 (72)
25     exp Genes, BRCA2/ or exp Genes, BRCA1/ or [brca1.mp](http://brca1.mp/). (10753)
26     exp BRCA2 Protein/ or [brca2.mp](http://brca2.mp/). (6557)
27     hereditary breast cancer*.mp. (781)
28     (HEREDITARY BREAST and OVARIAN CANCER SYNDROME).mp. [mp=title, abstract, original title, name of substance word, subject heading word, keyword heading word, protocol supplementary concept word, rare disease supplementary concept word, unique identifier] (157)
29     exp BRCA1 Protein/ (3477)
30     ("breast cancer 1" or breast cancer type 1).mp. [mp=title, abstract, original title, name of substance word,
subject heading word, keyword heading word, protocol supplementary concept word, rare disease supplementary concept word, unique identifier] (516)
31     ("breast cancer 2" or breast cancer type 2).mp. (279)
32     genetic [predisposition.mp](http://predisposition.mp/). or exp Genetic Predisposition to Disease/ (93864)
33     exp Breast Neoplasms/ or (ovarian carcinoma* or breast carcinoma* or ovarian cancer* or ovarian neoplasm* or breast cancer* or breast neoplasm*).mp. (314845)
34     32 and 33 (6302)
35     25 or 26 or 27 or 28 or 30 or 31 or 34 (16254)
36     ((family history or familial*) adj1 (ovarian carcinoma* or breast carcinoma* or ovarian cancer* or ovarian
neoplasm* or breast cancer* or breast neoplasm*)).mp. [mp=title, abstract, original title, name of substance word, subject heading word, keyword heading word, protocol supplementary concept word, rare disease supplementary concept word, unique identifier] (1317)
37     35 or 36 (16680)
38     (hormone adj2 therap*).mp. [mp=title, abstract, original title, name of substance word, subject heading word, keyword heading word, protocol supplementary concept word, rare disease supplementary concept word, unique identifier] (26211)
39     exp Hormone Replacement Therapy/ (20628)
40     exp Progesterone Congeners/ or exp Estrogens/ or [hrt.mp](http://hrt.mp/). (180437)
41     exp Progesterone/ or (progesterone adj1 (replacement* or therap*)).mp. (63082)
42     38 or 39 or 40 or 41 (204693)
43     exp Ovariectomy/ (20317)
44     (oophorectom* or ovariectom* or oophorotom* or ovarectom* or ovariotom* or ovarotom* or ovary amputation* or ovary resection* or salpingooophorectom*).mp. [mp=title, abstract, original title, name of substance word, subject heading word, keyword heading word, protocol supplementary concept word, rare disease supplementary concept word, unique identifier] (35698)
45     (female adj castration*).mp. [mp=title, abstract, original title, name of substance word, subject heading word, keyword heading word, protocol supplementary concept word, rare disease supplementary concept word, unique identifier] (27)
46     (BPSO or BOO or BSO or BPO or RRSO).mp. (3518)
47     43 or 44 or 45 or 46 (38969)
48     37 and 42 and 47 (72)

**Database: Embase <1974 to 2014 Week 10>**Search Strategy:
--------------------------------------------------------------------------------
1     exp BRCA2 protein/ or exp BRCA1 protein/ (10834)
2     (BCRA* or breast cancer type 1 or breast cancer type 2).mp. (381)
3     familial cancer/ or exp genetic predisposition/ (85814)
4     exp breast tumor/ (347062)
5     exp ovary cancer/ (73287)
6     ((hereditary or genetic predisposition* or familial or family history) adj1 (breast cancer* or breast neoplasm* or breast carcinoma* or ovar* cancer* or ovar* neoplasm* or ovar* carcinoma*)).mp. (8677)
7     (breast cancer* or breast neoplasm* or breast carcinoma* or ovar* cancer* or ovar* neoplasm* or ovar*
carcinoma*).mp. (393377)
8     3 and (4 or 5 or 7) (7590)
9     1 or 2 or 6 or 8 (21148)
10     hormone replacement therap*.mp. or exp hormone substitution/ (50266)
11     hormone substitution*.mp. (29283)
12     (progesterone adj1 (replacement* or therap*)).mp. [mp=title, abstract, subject headings, heading word, drug trade name, original title, device manufacturer, drug manufacturer, device trade name, keyword] (4418)
13     exp estrogen/ (223236)
14     exp progesterone/ (77616)
15     [hrt.mp](http://hrt.mp/). (11036)
16     10 or 11 or 12 or 13 or 14 or 15 (285069)
17     exp ovariectomy/ (28267)
18     (oophorectom* or ovariectom* or oophorotom* or ovarectom* or ovariotom* or ovarotom* or ovary amputation* or ovary resection* or salpingooophorectom*).ti,ab. (34545)
19     (female adj castration*).ti,ab. (29)
20     (BPSO or BOO or BSO or BPO or RRSO).ti,ab. (4567)
21     17 or 18 or 19 or 20 (46318)
22     9 and 16 and 21 (204)
***************************

SCOPUS Searched Feb 25, 2014

(TITLE-ABS-KEY(**hrt** OR **"hormone therap*"** OR **"hormone substitution"** OR **"hormone replace*"** OR **progesterone*** OR **estrogen**)) AND ((TITLE-ABS-KEY(**bcra*** OR **"hereditary breast cancer"** OR **"breast cancer 1"** OR**"breast cancer type 1"** OR **"breast cancer 2"** OR **"breast cancer type 2"**)) OR ((TITLE-ABS-KEY((**"ovarian carcinoma*"** OR **"breast carcinoma*"** OR **"ovarian cancer*"** OR **"ovarian neoplasm*"** OR **"breast cancer*"** OR **"breast neoplasm*"**) **.**) AND TITLE-ABS-KEY(**"family history"** OR **familial** OR **"genetic predispos*"**))) OR ((TITLE-ABS-KEY(**bcra*** OR **"hereditary breast cancer"** OR **"breast cancer 1"** OR **"breast cancer type 1"** OR **"breast cancer 2"** OR **"breast cancer type 2"**)) OR ((TITLE-ABS-KEY((**"ovarian carcinoma*"** OR **"breast carcinoma*"** OR **"ovarian cancer*"** OR **"ovarian neoplasm*"** OR **"breast cancer*"** OR **"breast neoplasm*"**)  AND TITLE-ABS-KEY(**"family history"** OR **familial** OR **"genetic predispos*"**))))) AND ((TITLE(**bpso** OR **boo** OR **bso** OR **bpo** OR **rrso**)) OR (TITLE(**oophorectom*** OR **ovariectom*** OR **oophorotom*** OR **ovarectom*** OR **ovariotom*** OR **ovarotom*** OR **"ovary amputation"**)) OR (TITLE-ABS-KEY(**"ovary resection*"** OR **salpingooophorectom***)) OR (TITLE-ABS-KEY(**"female castration*"**)))

**CINAHL March 21, 2014**


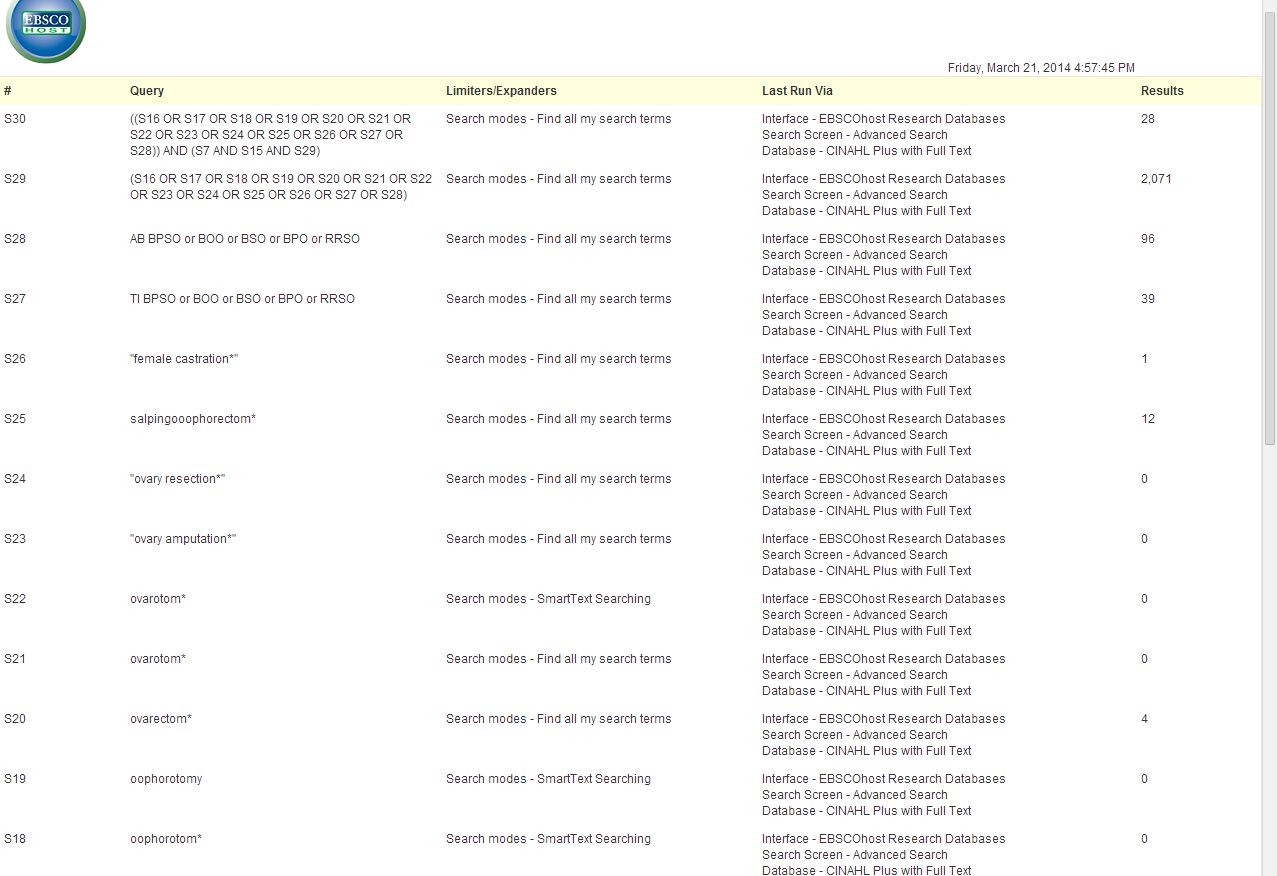


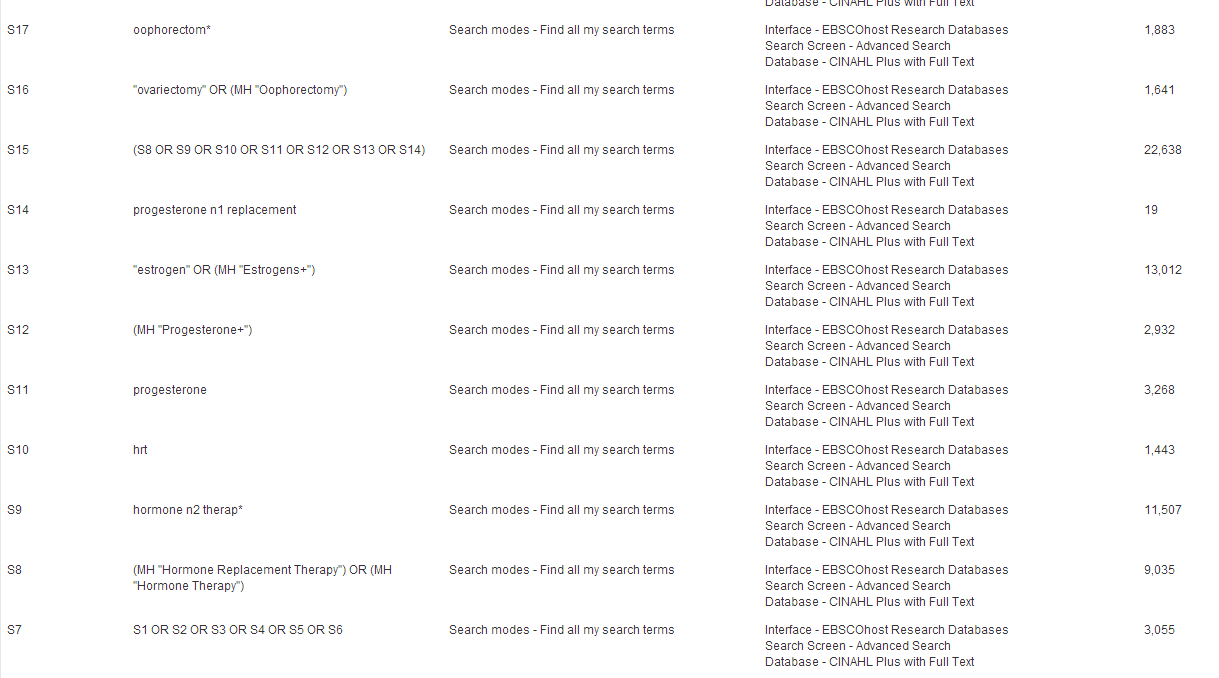


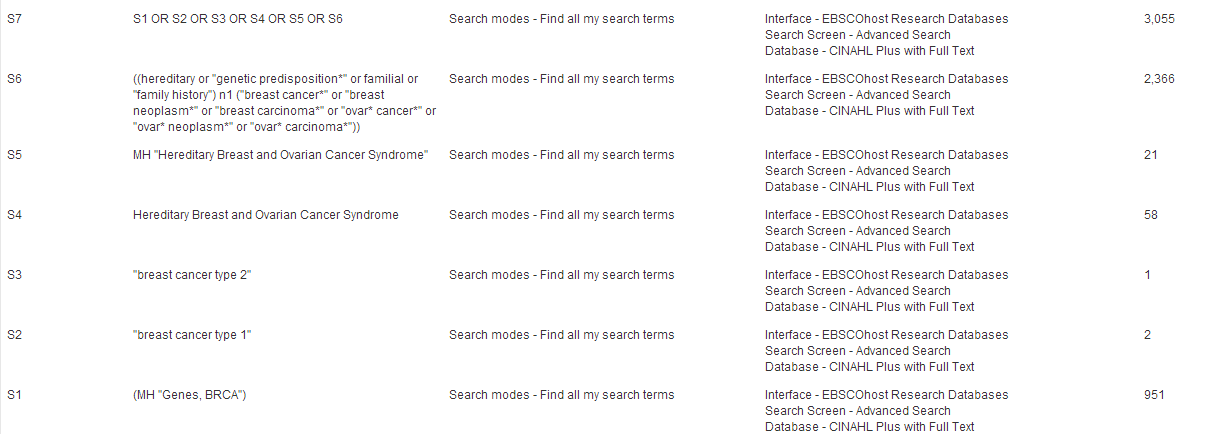


**Lilacs Searched March 21, 2014**


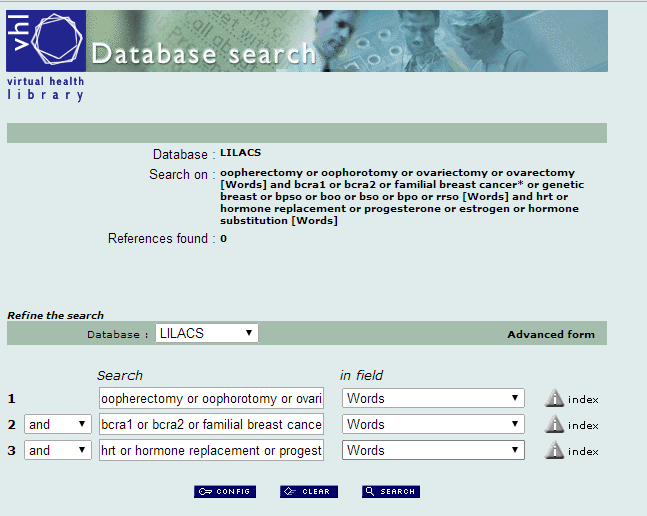


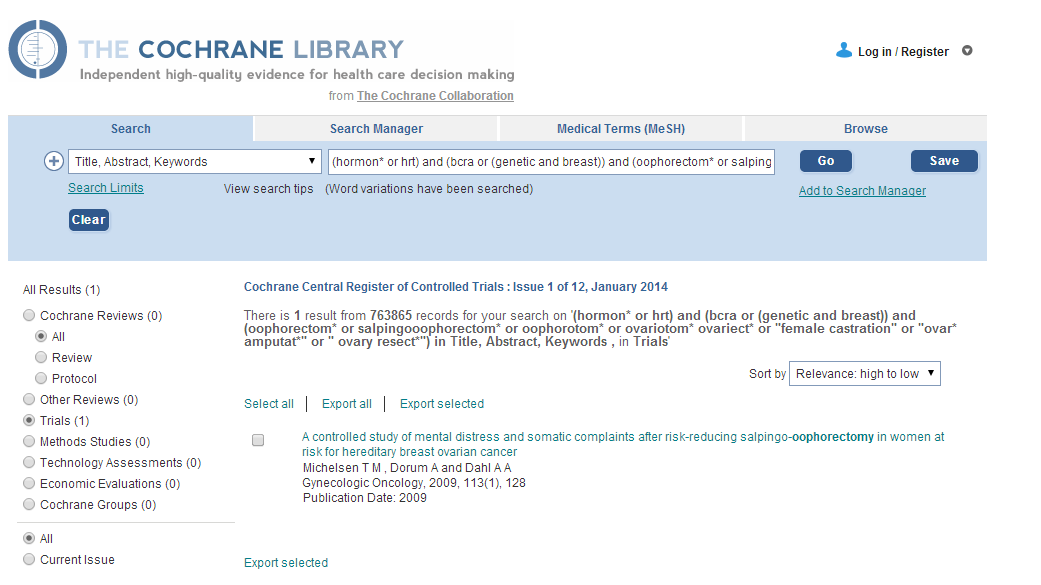


**Proquest Dissertations and Theses Searched March 21, 2014**

[**all(bpso OR boo OR bso OR bpo OR rrso OR oophorectom* OR ovariectomy* OR oophorotom* OR ovariectomy* OR ovariotomy* OR ovariotomy* OR "ovary amputation" OR "ovary resection*" OR salpingooophorectom* "female castration*") OR all((("ovarian carcinoma*" OR "breast carcinoma*" OR "ovarian cancer*" OR "ovarian neoplasm*" OR "breast cancer*" OR "breast neoplasm*") AND ("family history" OR familial OR "genetic predispos*"))) AND all(bcra* OR "hereditary breast cancer" OR "breast cancer 1" OR "breast cancer type 1" OR "breast cancer 2" OR "breast cancer type 2") AND all(hrt OR "hormone therap*" OR "hormone substitution" OR "hormone replace*" OR progesterone* OR estrogen*)**](http://search.proquest.com/results.displayspellingsuggestions_0:dospellingsearch?site=pqdtft&t:ac=E9935094C11E4293PQ/1) = 1

PsycINFO Searched March 21, 2014

Database: PsycINFO <1987 to March Week 3 2014>
Search Strategy:
--------------------------------------------------------------------------------
1     [bcra.mp](http://bcra.mp/). (1)
2     (("ovarian carcinoma*" or "breast carcinoma*" or "ovarian cancer*" or "ovarian neoplasm*" or "breast cancer*" or "breast neoplasm*") adj2 ("family history" or familial or "genetic predispos*")).mp. [mp=title, abstract, heading word, table of contents, key concepts, original title, tests & measures] (233)
3     ("hereditary breast cancer" or "breast cancer 1" or "breast cancer type 1" or "breast cancer 2" or "breast cancer type 2").mp. [mp=title, abstract, heading word, table of contents, key concepts, original title, tests & measures] (98)
4     1 or 2 or 3 (328)
5     (bpso or boo or bso or bpo or rrso or oophorectom* or ovariectom* or oophorotom* or ovarectom* or ovariotom* or ovarotom* or "ovary amputation" or "ovary resection*" or salpingooophorectom* or "female castration*").mp. [mp=title, abstract, heading word, table of contents, key concepts, original title, tests & measures] (2413)
6     (hrt or "hormone therap*" or "hormone substitution" or "hormone replace*" or progesterone* or estrogen*).mp. [mp=title, abstract, heading word, table of contents, key concepts, original title, tests & measures] (8247)
7     4 and 5 and 6 (1)
