# Supplementary material for: The effect of hormone therapy on quality of life and breast cancer risk after risk-reducing salpingo-oophorectomy: a systematic review
Source: BMC Womens Health. 2017 Mar 21;17:22. doi: 10.1186/s12905-017-0370-6 (PMC5359830; doi:10.1186/s12905-017-0370-6)
Supplement: Additional file 2: — A Search Strategy for Grey Literature: An Update. The document provides an update of the grey literature search performed in July, 2016, on SCOPUS, Proquest Dissertations and Theses, Clinical trial registries, Web of Science, and Google Scholar. (DOCX 22 kb) [file 12905_2017_370_MOESM2_ESM.docx]

**Additional file 2: A Search Strategy for Grey Literature: An Update**

SCOPUS Update search July 22, 2016

( ( ( TITLE ( **bpso**  OR  **boo**  OR  **bso**  OR  **bpo**  OR  **rrso** ) )  OR  ( TITLE ( **oophorectom***  OR  **ovariectom***  OR  **oophorotom***  OR  **ovarectom***  OR  **ovariotom***  OR  **ovarotom***  OR  **"ovary amputation"** ) )  OR  ( TITLE-ABS-KEY ( **"ovary resection*"**  OR **salpingooophorectom*** ) )  OR  ( TITLE-ABS-KEY ( **"female castration*"** ) ) ) )  AND  ( ( ( ( TITLE-ABS-KEY ( **hrt**  OR  **"hormone therap*"**  OR  **"hormone substitution"**  OR  **"hormone replace*"**  OR  **progesterone***  OR  **estrogen** ) ) )  AND  ( ( TITLE-ABS-KEY ( **bcra***  OR **"hereditary breast cancer"**  OR  **"breast cancer 1"**  OR  **"breast cancer type 1"**  OR  **"breast cancer 2"**  OR  **"breast cancer type 2"** ) ) ) )  OR  ( ( ( TITLE-ABS-KEY ( **"ovarian carcinoma*"**  OR  **"breast carcinoma*"**  OR  **"ovarian cancer*"**  OR  **"ovarian neoplasm*"**  OR **"breast cancer*"**  OR  **"breast neoplasm*"** ) ) )  AND  ( ( TITLE-ABS-KEY ( **"family history"**  OR  **familial**  OR  **"genetic predispos*"** ) ) ) )  OR  ( ( ( ( TITLE-ABS-KEY ( **bcra***  OR  **"hereditary breast cancer"**  OR  **"breast cancer 1"**  OR  **"breast cancer type 1"**  OR  **"breast cancer 2"**  OR  **"breast cancer type 2"** ) ) )  OR  ( ( TITLE-ABS-KEY ( **"ovarian carcinoma*"**  OR  **"breast carcinoma*"**  OR  **"ovarian cancer*"**  OR  **"ovarian neoplasm*"**  OR  **"breast cancer*"**  OR  **"breast neoplasm*"** ) ) ) )  AND  ( ( TITLE-ABS-KEY ( **"family history"**  OR **familial**  OR  **"genetic predispos*"** ) ) ) ) )  87 from 2014 – 2016)

ProQuest dissertation

[**(all(bpso OR boo OR bso OR bpo OR rrso OR oophorectom* OR ovariectomy* OR oophorotom* OR ovariectomy* OR ovariotomy* OR ovariotomy* OR "ovary amputation" OR "ovary resection*" OR salpingooophorectom* "female castration*") OR all((("ovarian carcinoma*" OR "breast carcinoma*" OR "ovarian cancer*" OR "ovarian neoplasm*" OR "breast cancer*" OR "breast neoplasm*") AND ("family history" OR familial OR "genetic predispose*")))) AND all(bcra* OR "hereditary breast cancer" OR "breast cancer 1" OR "breast cancer type 1" OR "breast cancer 2" OR "breast cancer type 2") AND all(hrt OR "hormone therapy*" OR "hormone substitution" OR "hormone replace*" OR progesterone* OR estrogen*)**](http://search.proquest.com/results.displayspellingsuggestions_0:dospellingsearch?site=pqdtglobal&t:ac=A7803BF434914182PQ/1) = 2 (but neither from 2014 or later so not exported)

Clinicaltrials.gov

 (hrt or hormone*) or (bpso or boo or bso or bpo or itso or oophorect* or ovariect*) and ("breast cancer*" or BCRA or famil* or genetic*) =0

Web of Science

 (bpso OR boo OR bso OR bpo OR rrso OR oophorectom* OR ovariectomy* OR oophorotom* OR ovariectomy* OR ovariotomy* OR ovariotomy* OR "ovary amputation" OR "ovary resection*" OR salpingooophorectom* "female castration") *AND* **TOPIC:**(BCRA or (("ovarian carcinoma" OR "breast carcinoma*" OR "ovarian cancer*" OR "ovarian neoplasm" OR "breast cancer" OR "breast neoplasm") and (hereditary or famil* or genetic))) *AND* **TOPIC:** (hrt OR "hormone therapy" OR "hormone substitution" OR "hormone replace" OR progesterone* OR estrogen*) = 14

Google Scholar -

brca oophorectomy* "hormone replacement" 2014 – 2016 Selected 107 titles
