# Supplementary material for: The effect of hormone therapy on quality of life and breast cancer risk after risk-reducing salpingo-oophorectomy: a systematic review
Source: BMC Womens Health. 2017 Mar 21;17:22. doi: 10.1186/s12905-017-0370-6 (PMC5359830; doi:10.1186/s12905-017-0370-6)
Supplement: Additional file 3: — The Effect of Hormone Therapy on Quality of Life and Breast Cancer Risk After Risk-Reducing Salpingo-oophorectomy: Meta-analyses of Pooled studies. The document provides a written description of the methods and the results of the meta-analyses conducted in our study. (DOCX 39 kb) [file 12905_2017_370_MOESM3_ESM.docx]

**Additional file 3: The Effect of Hormone Therapy on Quality of Life and Breast Cancer Risk After Risk Reducing Salpingo-oophorectomy: Meta-analysis of Pooled studies**

**Data synthesis**

Studies addressing the same outcome were assessed qualitatively for clinical and methodological heterogeneity. In case of inconsistencies, data were synthesized using evidence summaries or tables. When sufficient homogeneity was demonstrated, outcome data were pooled quantitatively via a meta-analysis. DerSimonian-Laird random effects model (accounting for part of the unexplained heterogeneity between studies) was used to calculate the pooled odds ratio (OR) and 95% confidence interval (CI) for discrete variables and weighted mean differences (WMD) and 95% CI for continuous variables. Heterogeneity in results of individual studies was assessed statistically using the I^2^ statistic, with an I^2^ >50% considered evidence for significant heterogeneity. Effect estimates were pooled irrespective of the extent of statistical heterogeneity. All statistical analyses were conducted using Review manager software (RevMan version 5.3.4; Copenhagen, Nordic Cochrane Centre, Cochrane Collaboration, 2008). Assessment to identify the risk of bias across studies was not performed because of the small number of studies included.

**Results**

*Quality of life (QOL)*

The WMD for the two homogenous studies showed improved QOL associated with HT use. The pooled estimate of WMD from random effect model was 3.27 (95% CI, 0.88-5.65, P <0.01, I2 = 0%)[^1^](#_ENREF_1)^,^ [^2^](#_ENREF_2)

*Breast cancer*

For this variable, no sufficient qualitative homogeneity was demonstrated across relevant studies to justify pooling of estimates; hence no meta-analysis was performed.

*Vasomotor symptoms*

The pooled OR for three studies showed that HT reduced the prevalence of hot flashes (OR = 0.38; 95%CI, 0.23-0.62, P<0.01, I2 = 0%).[^1-3^](#_ENREF_1)The pooled OR for two studies that reported night sweats showed a reduction with HT (OR = 0.42; 95%CI, 0.24-0.74, P<0.01, I2 = 0%).[^1^](#_ENREF_1)^,^ [^2^](#_ENREF_2)

*Sexual function*

Pooled estimates failed to show improvement with HT in any of the measured aspects of sexual activity (pleasure, discomfort, habit or libido) in the two homogenous studies that reported this outcome.[^2^](#_ENREF_2)^,^ [^3^](#_ENREF_3) WMD for pleasure was 0.62; 95%CI, -0.27-1.51, P = 0.17, I2 = 0%. WMD for discomfort was 1.12; 95%CI, -0.36-2.61, P = 0.14, I2 = 0%. WMD for habit was 0.12, 95%CI, -0.02-0.27, P = 0.09, I2 = 0%. OR for libido was 0.67; 95%CI, 0.40-1.14, I2 = 0%.

*Vaginal dryness*

Pooled analysis showed a lower prevalence of vaginal dryness in women taking HT, in contrast to the two individual studies (OR = 0.48; 95%CI, (0.26-0.87), P = 0.02, I2 = 0%).[^1^](#_ENREF_1)^,^ [^2^](#_ENREF_2)

*Bone Loss*

HT users had less bone loss compared to non-users in the 3 studies included in the pooled analysis (OR = 0.45; 95%CI, 0.23-0.86, P = 0.02, I2 = 0%).[^1^](#_ENREF_1)^,^ [^4^](#_ENREF_4)^,^ [^5^](#_ENREF_5)

**References**

1. Challberg J, Ashcroft L, Lalloo F, Eckersley B, Clayton R, Hopwood P, et al. Menopausal symptoms and bone health in women undertaking risk reducing bilateral salpingo-oophorectomy: significant bone health issues in those not taking HRT. Br J Cancer. 2011 Jun 28;105(1):22-7.

2. Madalinska JB, van Beurden M, Bleiker EM, Valdimarsdottir HB, Hollenstein J, Massuger LF, et al. The impact of hormone replacement therapy on menopausal symptoms in younger high-risk women after prophylactic salpingo-oophorectomy. J Clin Oncol. 2006 Aug 1;24(22):3576-82.

3. Finch A, Metcalfe KA, Chiang JK, Elit L, McLaughlin J, Springate C, et al. The impact of prophylactic salpingo-oophorectomy on menopausal symptoms and sexual function in women who carry a BRCA mutation. Gynecol Oncol. 2011 Apr;121(1):163-8.

4. Garcia C, Lyon L, Conell C, Littell RD, Powell CB. Osteoporosis risk and management in BRCA1 and BRCA2 carriers who undergo risk-reducing salpingo-oophorectomy. Gynecol Oncol. 2015 Sep;138(3):723-6.

5. Chapman JS, Powell CB, McLennan J, Crawford B, Mak J, Stewart N, et al. Surveillance of survivors: follow-up after risk-reducing salpingo-oophorectomy in BRCA 1/2 mutation carriers. Gynecol Oncol. 2011 Aug;122(2):339-43.
