# Supplementary material for: The effect of hormone therapy on quality of life and breast cancer risk after risk-reducing salpingo-oophorectomy: a systematic review
Source: BMC Womens Health. 2017 Mar 21;17:22. doi: 10.1186/s12905-017-0370-6 (PMC5359830; doi:10.1186/s12905-017-0370-6)
Supplement: Additional file 4: — The results of full-text screening of eligible articles and the reason for exclusion whenever this may apply. (DOCX 19 kb) [file 12905_2017_370_MOESM4_ESM.docx]

| **Ref work ID** | **First Author** | **Decision** | **Reason for exclusion** |
| --- | --- | --- | --- |
| 191-3 | Alanbay | 0 | Study design doesn’t meet eligibility criteria (Book review) |
| 234-1 | Antoine | 0 | Study design doesn’t meet eligibility criteria (Review paper) |
| 50-1 | Armstrong | 0 | Study design doesn’t meet eligibility criteria (Markov decision analysis) |
| 152-3 | Barlin | 0 | Study design doesn’t meet eligibility criteria (Review paper) |
| 696-1 | Barry | 0 | Study design doesn’t meet eligibility criteria  (Review paper) |
| 128-3 | Breda | 0 | Study design doesn’t meet eligibility criteria (Review paper) |
| 212-1 | Castiqlione | 0 | Study design doesn’t meet eligibility criteria (Review paper) |
| 10-1 | Challberg | 1 |  |
| 167-1 | Chang-Claude | 0 | Effect of HT on risk of breast cancer not assessed. Only studied effect of age at menarche; time of breast mitotic activity from menarche till first pregnancy and menopause; age at menopause; menopause status; and type of menopause on breast cancer risk among carriers of BRCA1/2 mutations |
| 9-1 | Chapman | 1 |  |
| 104-3 | Cibula | 0 | Study design doesn’t meet eligibility criteria (Meta-analysis). Also, study population doesn’t fit eligibility criteria – no RRSO performed |
| 107-3 | Cui | 0 | Study population doesn’t fit eligibility criteria (No BRCA carriers and not all surgically menopausal women are high risk) |
| 731-1 | Domchek | 0 | Effect of HT on mortality among BPSO not assessed (Intervention BPSO and comparator non-BPSO) |
| 623-1 | Dorum | 0 | Effect of HT on Metabolic syndrome and Framingham risk score among BPSO not assessed (Intervention BPSO and comparator non-BPSO) |
| 766-1 | Eisen | 1 |  |
| 268-1 | Eltabbakh | 0 | Outcome doesn’t meet eligibility criteria (Bleeding with HT) |
| 11-1 | Finch | 1 |  |
| 802-1 | Foulkes | 0 | Effect of HT on risk of breast cancer not assessed. Studied effect of mutation, age and type of breast cancer on estrogen receptor status of breast cancer. |
| 22--1 | Gabriel | 1 |  |
| 1-2 | Garcia | 1 |  |
| 153-3 | Guidozzi | 0 | Study design doesn’t meet eligibility criteria (Review paper) |
| 777-1 | Haile | 0 | Effect of oral contraceptive on breast cancer among BPSO patients not assessed (population included BRCA 1 and 2 mutations not necessarily had BPSO) |
| 672-1 | Hallowell | 0 | Study design doesn't fit eligibility criteria (Editorial) |
| 48-1 | Hallowell | 0 | Study design doesn’t meet eligibility criteria (Descriptive/qualitative) |
| 3-2 | Heiniger | 1 |  |
| 133-3 | Johansen | 1 |  |
| 753-1 | King | 0 | Effect of BPSO and HT on risk of breast cancer not assessed (looked at lifetime risk of breast cancer among BRCA 1 and 2 mutations and effect of multiple family history of risk increase…as well as other non-genetic predispositions to breast cancer risk increase |
| 270-1 | Kontoravids | 0 | Outcome doesn’t meet eligibility criteria  (Rates of ovarian cancer following hysterectomies and oophorectomies) |
| 134-3 | Kotsopoulos | 1 |  |
| 765-1 | Laki | 0 | Study design doesn’t fit eligibility criteria (non-analytical no comparator). Also effect of HT on breast cancer risk not assessed |
| 33-3 | Levy-Lahad | 0 | Study design doesn’t meet eligibility criteria (Review paper) |
| 119-3 | Lorenz | 0 | Effect of HT on sexual activity post RRSO was not assessed. HT was included in the statistical model to control for its potential confounding effect on sexual activity and other psychosocial variables |
| 34-1 | Madalinska | 1 |  |
| 774-1 | Madalinska | 0 | Effect of HT on quality of life not assessed. (BPSO is intervention and comparator is gynecologic screening) |
| 61-1 | Meiser | 0 | Study design doesn’t meet eligibility criteria (Qualitative study) |
| 798-1 | Metcalfe | 0 | Effect of HT on breast cancer not assessed. Effect of HT on contralateral breast cancer not assessed |
| 796-1 | Metcalfe | 0 | Effect of HT on breast cancer not assessed. Only looked at predictors of risk of contra-lateral breast cancer (effect of HT not assessed though) |
| 132-1 | Michelsen | 0 | Effect of HT on anxiety and depression among BPSO patients not assessed-analysis done for whole group (RRSO and controls (non-BPSO) |
| 119-1 | Michelsen | 1 |  |
| 669-1 | Moller | 0 | Effect of HT on breast cancer risk not assessed (only survival with stage at diagnosis, BRCA status, and oophorectomy) |
| 800-1 | Narod | 0 | Effect of oral contraceptives on breast cancer was assessed for BRCA mutation carriers only. Type of menopause (natural/BPSO) was not measured or looked at. |
| 67-1 | Pines | 0 | Study design doesn’t meet eligibility criteria  (Case report) |
| 42-1 | Rebbeck | 1 |  |
| 727-1 | Rebbeck | 0 | Effect of HT on risk of breast cancer among BPSO patients not assessed |
| 735-1 | Roukos | 0 | Study design doesn’t meet eligibility criteria (Editorial) |
| 178-3 | Schrijver | 0 | Study design doesn’t meet eligibility criteria (Review paper) |
| 168-3 | Schuler-Toprak | 0 | Study design doesn’t meet eligibility criteria (Systematic review) |
| 729-1 | Struewing | 0 | Effect of HT on risk of breast cancer among BPSO patients not assessed. Only looked at incidence of breast cancer post-oophorectomy |
| 761-1 | Tiller | 0 | Study design doesn’t meet eligibility criteria (Qualitative study) |
| 159-3 | Tucker | 1 |  |
| 801-1 | Ursin | 0 | Effect of oral contraceptives on breast cancer was assessed for BRCA mutation carriers only. Type of menopause (natural/BPSO) was not measured or looked at |

Exclude “0” Include”1”

**Legend:**

|  | Study design doesn’t meet eligibility criteria |
| --- | --- |
|  | Effect of intervention not assessed |
|  | Outcome doesn’t fit eligibility criteria |
|  | Study population doesn’t fit eligibility criteria |
