# Supplementary material for: The effect of hormone therapy on quality of life and breast cancer risk after risk-reducing salpingo-oophorectomy: a systematic review
Source: BMC Womens Health. 2017 Mar 21;17:22. doi: 10.1186/s12905-017-0370-6 (PMC5359830; doi:10.1186/s12905-017-0370-6)
Supplement: Additional file 5: — A Checklist summarizing compliance with MOOSE guidelines. The attached document is a checklist highlighting the compliance of our systematic review with the quality criteria specified by MOOSE guidelines. (DOC 27 kb) [file 12905_2017_370_MOESM5_ESM.doc]

**Additional file 5: A Checklist summarising compliance with MOOSE guidelines**

| **Reporting background should include** | |
| --- | --- |
| Problem definition | Yes |
| Hypothesis statement | Yes |
| A statement of objectives that include the study population, the condition of interest, the exposure or intervention, and the outcome(s) considered | Yes |
| **Reporting of search strategy should include** | |
| Qualifications of searches (e.g. librarians and investigators) | Yes |
| Search strategy, including time period included in the synthesis and keywords | Yes |
| Effort to include all available studies, including contact with authors | Yes |
| Databases and registries searched | Yes |
| Search software used, name and version, including special features (appendix) | Yes |
| Use of hand searching (e.g. reference lists of obtained articles) – also electronic citation search | Yes |
| List of citations located and those excluded including justification | Yes |
| Method of addressing articles published in languages other than English | N/A |
| Method of handling abstracts and unpublished studies | None found |
| Description of any contact with authors | Yes |
| **Reporting methods should include** | |
| Description of relevance or appropriateness of studies assembled for assessing the hypothesis to be tested | Yes |
| Rationale for the selection and coding of data (eg, sound clinical principles or convenience) | Yes |
| Documentation of how data were classified and coded (eg, multiple raters, blinding, and interrater reliability) | Yes |
| Assessment of confounding (eg, comparability of cases and controls in studies where appropriate) | Yes |
| Assessment of study quality, including blinding of quality assessors; stratification or regression on possible predictors of study results | Yes |
| Assessment of heterogeneity | Yes |
| Description of statistical methods (eg, complete description of fixed or random effects models, justification of whether the chosen models account for predictors of study results, dose-response models, or cumulative meta-analysis) in sufficient detail to be replicated | Yes |
| Provision of appropriate tables and graphics | Yes |
| **Reporting of results should include** | |
| Graphic summarizing individual study estimates and overall estimate | Yes |
| Table giving descriptive information for each study included | Yes |
| Results of sensitivity testing (eg, subgroup analysis) | N/A |
| Indication of statistical uncertainty of findings | Yes |
| **Reporting of discussion should include** | |
| Quantitative assessment of bias (eg, publication bias) | N/A |
| Justification for exclusion (eg, exclusion of non–English-language citations) | Yes |
| Assessment of quality of included studies | Yes |
| **Reporting of conclusions should include** | |
| Consideration of alternative explanations for observed results | Yes |
| Generalization of the conclusions (ie, appropriate for the data presented and within the domain of the literature review) | Yes |
| Guidelines for future research | Yes |
| Disclosure of funding source | Yes |
